# Saussureae Involucratae Herba (Snow Lotus): Review of Chemical Compositions and Pharmacological Properties

**DOI:** 10.3389/fphar.2019.01549

**Published:** 2020-01-14

**Authors:** Guowei Gong, Jing Huang, Yang Yang, Baohui Qi, Guangyi Han, Yuzhong Zheng, Huan He, Kelvin Chan, Karl WK Tsim, Tina TX Dong

**Affiliations:** ^1^ Department of Biological Engineering, Zunyi Medical University, Zhuhai, China; ^2^ College of Environmental and Biological Engineering, Putian University, Putian, China; ^3^ Gansu Institute for Drug Control, Lanzhou, China; ^4^ Department of Biology, Hanshan Normal University, Chaozhou, China; ^5^ School of Pharmacy & Biomolecular Sciences, Liverpool John Moores University, Liverpool, United Kingdom; ^6^ Shenzhen Key Laboratory of Edible and Medicinal Bioresources, HKUST Shenzhen Research Institute, Shenzhen, China; ^7^ Division of Life Science and Center for Chinese Medicine, The Hong Kong University of Science and Technology, Hong Kong, Hong Kong

**Keywords:** Saussureae Involucratae Herba, traditional Chinese medicine, traditional Uyghur medicine, herbal medicine, Snow lotus

## Abstract

Saussureae Involucratae Herba is the dried ground part of *Saussurea involucrata* (Kar. et Kir.) Sch.-Bip, which is also named as “Snow lotus” and being used in traditional Uyghur and/or Chinese medicine. This rare herb can be found at 4,000 m elevation in western part of Tianshan Mountain, Xinjiang China. According to China Pharmacopoeia (2015), the major pharmaceutical values of “Snow lotus” (Xuě liánhuā in Chinese) are alleviating rheumatoid arthritis, accelerating blood circulation and mitigating other “cold” syndromes. Traditionally, the clinical application of “Snow lotus” includes the treatments in inflammation-associated disorder, blood circulation acceleration and heat and dampness elimination. Recent studies suggested that *“*Snow lotus” possessed therapeutic effects associating with anti-cancer, anti-oxidation, adipogenesis suppression and neuroprotection activities, which were proposed to be related with its bioactive constitutes, i.e. acacetin, hispidulin, and rutin. In the present review, we aim to summarize pharmacological effects and underlying cell signaling pathways of “Snow lotus” in treating various medical problems.

## Introduction

The history of Traditional Uyghur medicine (TUM) spans over 2,500 years and is still being practiced today (Upur et al., 2011). Ghazi Bay recorded fennel, senna, salt and other 312 types of TUM herbs, as well as its therapeutic functions, in the book of “Ghazi Bay medicinal book”, written in about 400 B.C. (Yishakejiang et al., 2005). The predominant concept of TUM is based on four unique elements of air, fire, water and soil, and each element is corresponding to specific humor, i.e. “phlegm”, “blood”, “yellow bile” and “black bile” (Shahabi et al., 2008). Uygur doctors believe that the occurrence of disease has close relationship with the destruction of temperament balance, which results in abnormal body fluid disequilibrium. The mainstay of health is to keep right ratio and precise balance of humors based on their quality and quantity. Therefore, adjusting and balancing body fluids are the fundamental principle for disorder treatments according to the TUM theory. Indeed, traditional Chinese medicine (TCM) and TUM share abundant similarities, and the major responsibilities of TUM and/or TCM are modulating health conditions, promoting health and having therapeutic strategies for specific diseases or symptom treatments. Nowadays, traditional medicine is often referring to complementary or alternative medicine. In developing countries, e.g. Asian and African, up to 80% of the population consumes herbal decoction in meeting the primary health care needs. A massive of ancient literatures, including book and pharmacopoeia, have recorded the existence of TCM and/or TUM prescriptions and their beneficial functions, disease preventions and clinical applications (Packer et al., 2004). Nevertheless, the metabolites, pharmaceutical values and action mechanisms of these TCM and/or TUM in modulating human health are still behind veil. This review provides some detailed information and pharmaceutical values of TUM and/or TCM herbal materials in general, using “Snow lotus” as an example.

**Figure 1:**
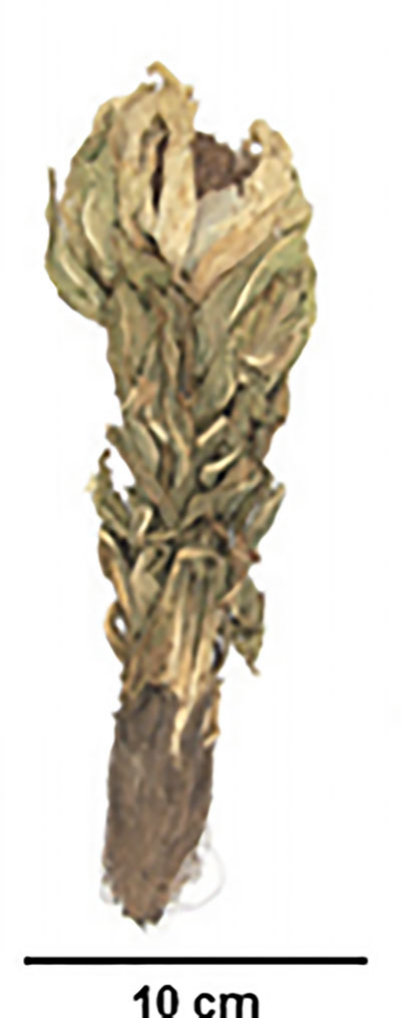
The pictures of Snow lotus. Saussureae Involucratae Herba is described in the HKCMMS Volume 8. http://www.cmd.gov.hk/hkcmms/vol8/pdf_e/Saussureae_Involucratae_Herba_v8_e.pdf.

**Figure 2:**
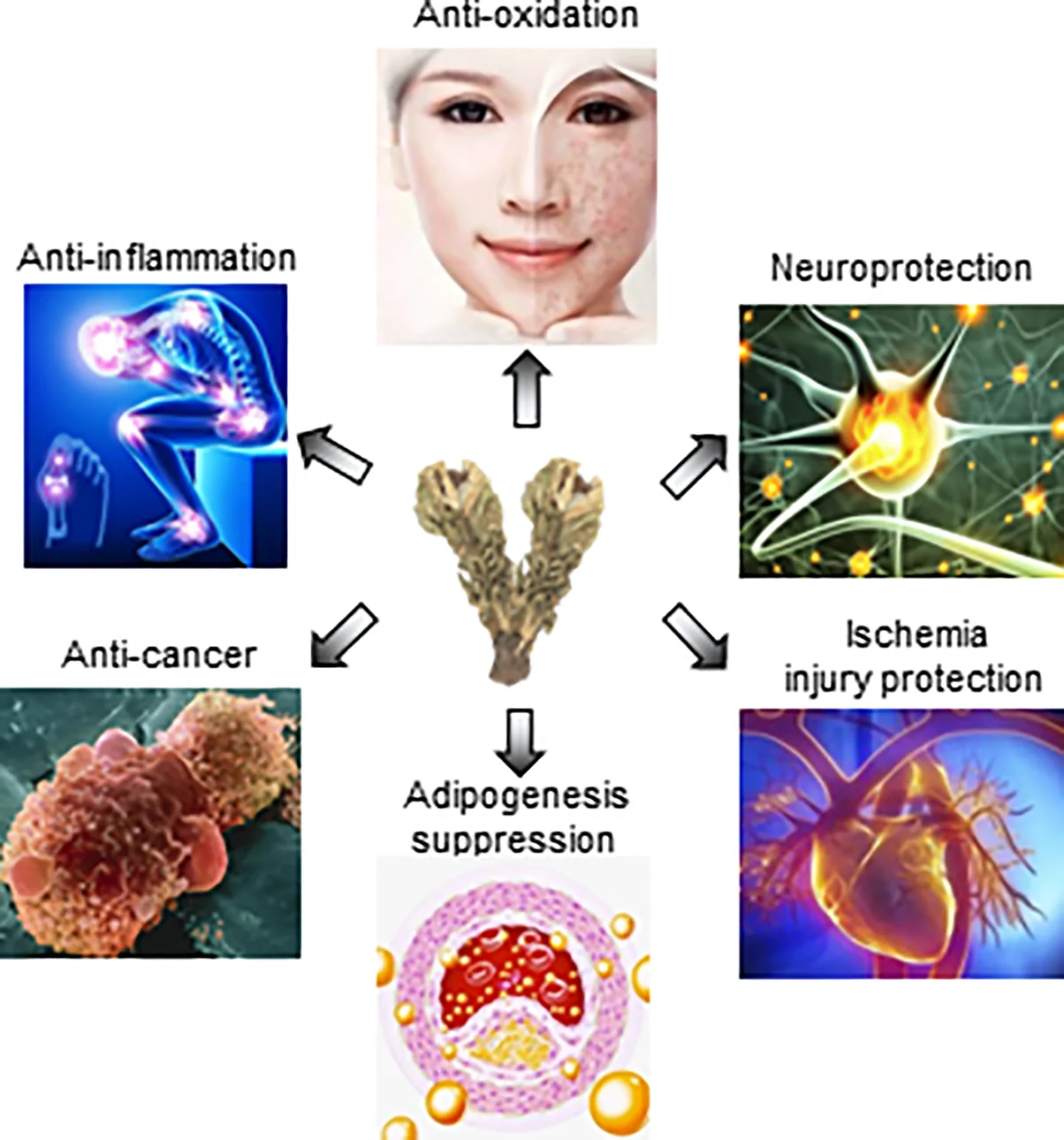
The pharmaceutical values of Snow lotus. Saussureae Involucratae Herba possesses anti-oxidative functions, neuroprotective effects, anti-inflammatory-induced diseases, anti-cancer, anti-obesity and ischemia injury protective pharmaceutical values.

**Figure 3:**
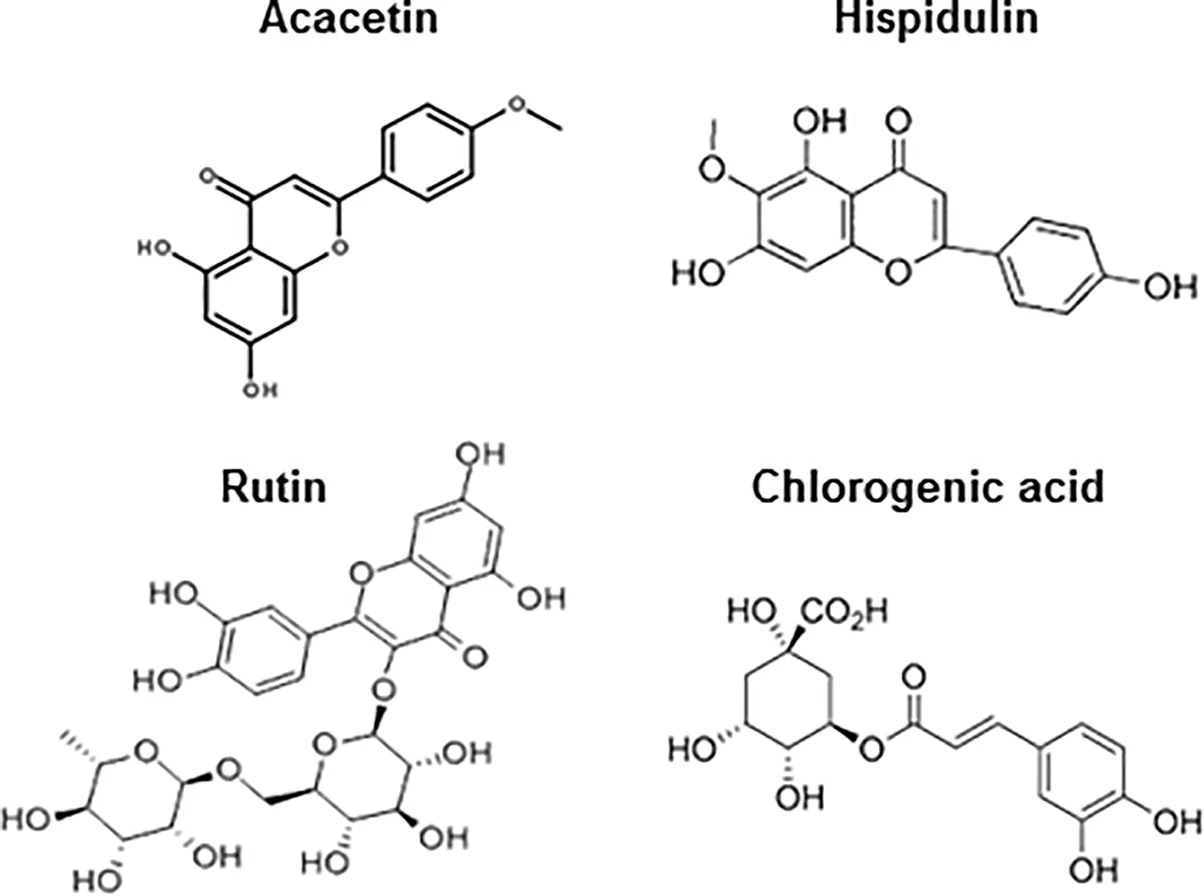
Chemical structures of major bioactive components isolated from Snow lotus. The chemical structures of acacetin, hispidulin, rutin and chlorogenic acid are shown here.

According to the China Pharmacopoeia (2015), “Snow lotus” is the dried ground part of *Saussurea involucrata* (Kar. et Kir.) Sch.-Bip (**Figure 1**), which is being used for inflammation-associated disorder treatments, such as rheumatoid arthritis, cancer, modulating lipid metabolism, and improving gynecological and reproductive problem, enhancing blood circulation and mitigation of other “cold” syndrome (Chik et al., 2015). The incidences of rheumatoid arthritis, gastric cancer and hepatic cancer are much less in Uygur population, as compared to other parts of China, probably have a close relationship with the common consumption of “Snow lotus” in Xinjiang area (Byambaragchaa et al., 2013; Yu et al., 2013; Chik et al., 2015; Wang et al., 2016). According to China Pharmacopeia (2015), the primary pharmaceutical values of “Snow lotus” is maintaining body homeostasis. **Figure 2** summarizes the possible functions of “Snow lotus”. The major bioactive components exhibited the clinical functions were reported to be acacetin, hispidulin and rutin (Chik et al., 2015). The structures of these chemicals are presently shown in **Figure 3**. Most of the “Snow lotus”-concentrated herbal decoctions are capable of mitigating “cold” syndromes or diseases both in male and female. This materia medica is one of the major ingredients found within **“**Snow lotus” capsule being sold in China, which is aiming for dysmenorrhoea treatment. Moreover, the water decoction having “Snow lotus”, *Lycii fructus* and *Angelica sinensis* Radix could promote the secretion of androgen, to enhance the sexual function for male patients, which therefore has been used for infertility treatment (Zhao, 1963).

## Botanical Classification

“Snow lotus” is a dicotyledonous plant, classified under Compositae. The commonly found “Snow lotus” is clustered into subgenus of *Amphilaena* and *Eriocovgne*. The subgenus of *Amphilaena* contains *S. involucrata, S．globosa*, S. *wettsteiniana*, S. *polycolea*, S. *uniflora*, S. *velutina*, S. *phaeantha*, S. *orgaadayi*, S. *tangutica*, S. *bracteata*, S. *erubescens*, S. *nigrescens*, S. *iodostegia*, S. *glandulosissima, and* S. *sikkimensi* (Chen et al., 2019). The subgenus of *Eriocovgne* includes *S．aster, S．glacialis, S．gnaphaloides, S. laniceps* and *S. medusa* (Zhai et al., 2009). However, *S. involucrata*, *S. laniceps*, and *S. medusa* are the most commonly used species in clinical applications (Yi et al., 2010).

The morphological features of *S. involucrata* show similarities with *S. laniceps* and *S. medusa* (Chen et al., 2014a). The microscopic characteristics, as determined by scanning electron microscopy (SEM), of these *Saussurea* species had been reported and identified: the pollen grains were the indicative markers showing their distinctive characteristics. The pollen grains of *S. laniceps* was sub-rounded, light yellow, covered with perforate and verruca warts. However, the pollen of *S. medusa* was bigger, and the outer surface was sculptured with dense spinules. *S. involucrata* had yellow pollen and wart on outer surface (Chen et al., 2014a). Recently, a new species of “Snow lotus”, named as *S. bogedaensis*, has been explored and reported in the eastern part of Tianshan Mountain (Chen and Wang, 2018). The genetic analysis showed that *S. bogedaensis* and *S. involucrata* had a close relationship, which might suggest that these two plants are deriving from a common ancestor (Chen and Wang, 2018). Moreover, *S. bogedaensis* shares similar pharmaceutical values with *S. involucrate*; hence, *S. bogedaensis* could act as an alternative to *S. involucrata* in clinical applications. Yi et al., 2010 conducted systematic experiments to demonstrate that *S. laniceps* was the most potent *Saussurea* species as the source of TCM, instead of *S. involucrata* or *S. medusa*.

## Biosynthesis of Secondary Metabolites

**Figure 4:**
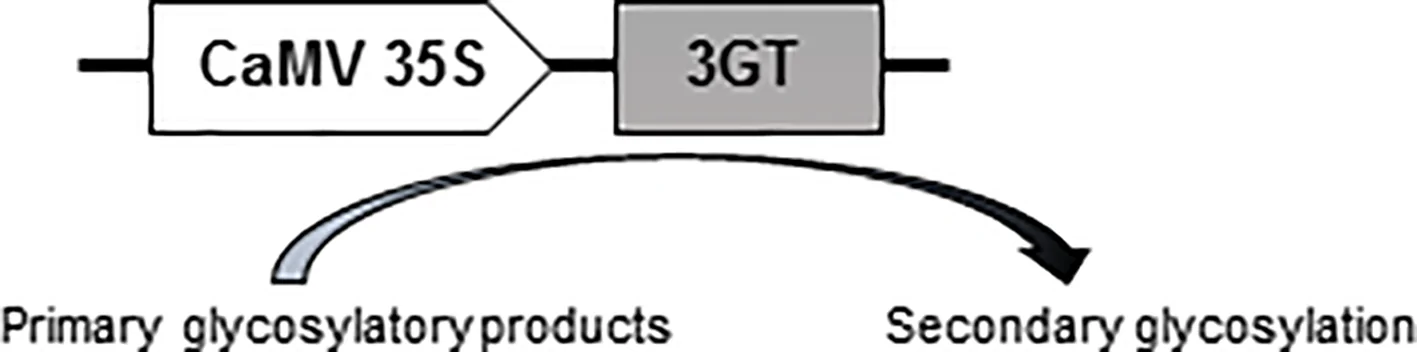
Flow chart of secondary metabolites. The employment of 3GT was used in *S. involucrata* to catalyze the formation of primary glycosylatory metabolites.

**Figure 5:**
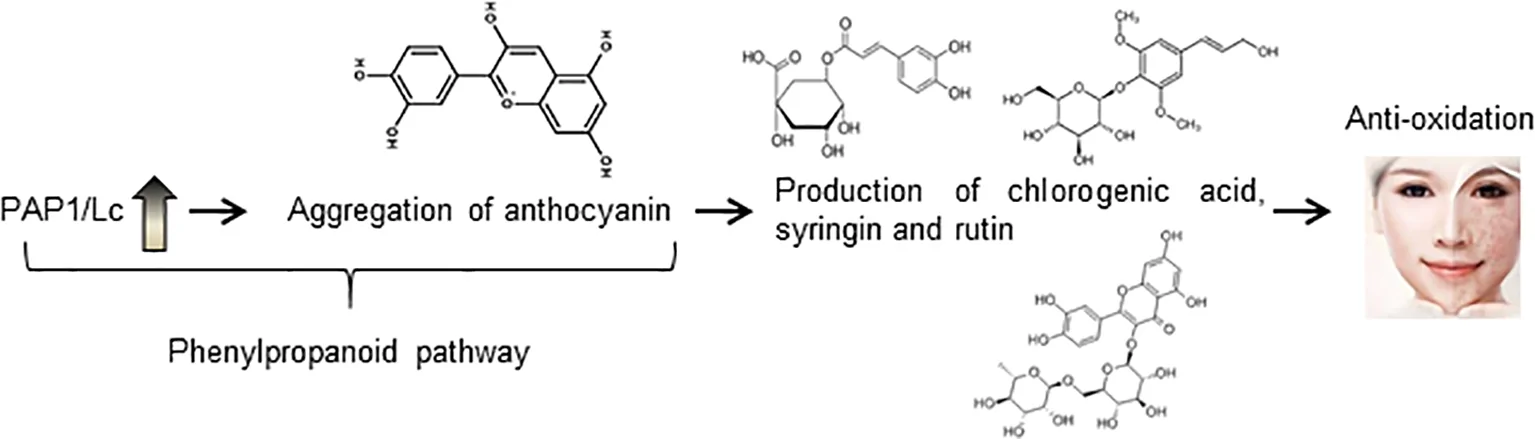
Flow chart of phenylpropanoid pathways. PAP1/Lc up-regulates the biosynthetic pathway of anthocyanin, and which has the mentioned anti-oxidative functions.

The full-length gene encoding flavonoid-3-O-glucosyltransferase (3GT) (GenBank Accession No. JN092127), cloned from *S. involucrata*, was isolated and employed to search for the biosynthesis of plant secondary metabolites (**Figure 4**). The transcripts of 3GT members were detected massively in leaves and callus of *S. involucrata*, and which were able to catalyze the secondary glycosylation products by transferring activated sugar donors to acceptors. The 3GT gene of *S. involucrata* was constructed under the control of a cauliflower mosaic virus (CaMV) 35S promoter, and then the homologous transformation was done by an agrobacterium rihizogenes-mediated transformation system (**Figure 4**) (Tang et al., 2012). Thus, a transgenic *S. involucrata* having over expression of 3GT gene was generated. Two major transcriptional factors, i.e. anthocyanin pigment 1 (PAP1) and leaf color (Lc), involved in the phenylpropanoid pathways are proposed to contribute regulatory functions of the biosynthetic pathways (Qiu et al., 2013). In the genetic modified plant, *S. involucrata*, the over expressed PAP1 and Lc were able to accumulate purple pigments by the massive aggregation of anthocyanin (Qiu et al., 2013). In parallel, the increased augmentations of chlorogenic acid, syringin, cyanrine and rutin in the transgenic herbal material were able to increase its anti-oxidative functions, as measured by ABTS (2,2’-azinobis-3-ethylbenzotiazo-line-6-sulfonic acid) and FRAP (ferric reducing anti-oxidant power) (**Figure 5**) (Qiu et al., 2010).

## Fungus and Host Plant

Fungal growth on *S. involucrata* has been reported to play unique roles. Endophytic fungi, responsible for species diversity and keeping the host fitness, were isolated and identified by morphological and molecular methods from *S. involucrata*. The isolated fungi are classified into 14 taxa, and most of them are belonging to *Cylindrocarpon* sp. (Lv et al., 2010). Some of *S. involucrata* plants contain dark pigment that is named as dark septate endophytic (DSE) fungus (Lv et al., 2010). The most predominant DSE fungus was DSE-37, as determined by the DNA fragment of internal transcribed spacer regions. The results indicated that DSE-37 was congeneric to *Mycocentrospora* (Wu et al., 2010). After over one-month cultivation of *S. involucrata* in the present of DSE-37, fungal hyphae were branched and twisted together and finally formed “hyphae nets” in epidermal layers, which was proposed to be the positive effect of DSE-37 on the root development. Besides, the content of rutin was much concentrated in DSE-37 positive *S. involucrata* plants as compared to the control, indicating DSE-37 was one of indispensable constituents in supporting the development of “Snow lotus” (Wu et al., 2010).

## Biological Activity of “Snow Lotus”

### Neuroprotection Effect

“Snow lotus” was found to have therapeutic effects against severe acute pancreatitis (SAP)-induced brain injury using *in vivo* tests. In SAP-induced rats, administration of the extract of “Snow lotus” decreased the mortality rate. In parallel, the serum levels of endothelin-1 (ET-1) and nitric oxide (NO) were significantly reduced, as compared to the control group (Wang et al., 2018). Meanwhile, the pathological changes in pancreas and brain were milder, as comparing to SAP-untreated rats. It is also found that PI3K/Akt signaling pathway was associated with development and progression of SAP-induced brain injury. The intraperitoneal injection of “Snow lotus” extract in SAP-treated rats induced the protein expression of PI3K/Akt (Wang et al., 2018), which should be an indication of therapeutic function of “Snow lotus” in brain damage (**Table 1**). In another investigation on “Snow lotus” extract using D-galactose-induced brain injury mice, promising therapeutic functions were observed. After 6-week of recovery with the herbal treatment in the injured mice, an enhancement of superoxide dismutase and glutathione peroxidase, as well as a decrease of lipid peroxidation, was revealed in the plasma (Yang et al., 2012). Furthermore, the ethyl acetate extract of “Snow lotus”-treated mice showed an improved behavioral performance in step-through passive avoidance task (Yang et al., 2012).

**Table 1:** Summary of neuro-protection functions of “Snow lotus” and/or its major biochemical.

Models	Parameters measured	Active components	Reference
*In vivo*	ET-1 and NO content in serum	“Snow lotus” extract	Wang et al., 2018
*In vivo*	Behavioral test	Ethyl acetate extract of “Snow lotus”	Yang et al., 2012
*In vivo*	Cytosolic free Ca^2+^ concentration	Acacetin	Lin et al., 2014
*In vivo/ in vitro*	Oxidative stress-induced damage, inflammatory responses and neuronal	Rutin	Qu et al., 2014
*In vivo*	Malondialdehyde	Rutin	Motamedshariaty et al., 2014
*In vivo*	AMPK and GSK3β activations	Hispidulin	Niu et al., 2014
*In vitro*	Neurotropic factors production	Rutin	Na et al., 2014
*In vitro*	Glutamate activation	“Snow lotus” extract	Lin et al., 2012a

Acacetin, an o-methylated flavone from “Snow lotus” or other Asteraceae family (Zhao et al., 2016b), was shown to be a neuroprotective agent by suppression of depolarization-evoked glutamate release and cytosolic free Ca^2+^ concentration in the hippocampal nerve terminals, which was hypothesized to be prevented by Cav2.2 (N-type) and Cav2.1 (P/Q-type) channel blockers (Lin et al., 2014). Rutin was a predominant bioactive constituent found within “Snow lotus”. Consumption of rutin was capable of alleviating the permanent bilateral common carotid artery occlusion (BCCAO) in rat model by revealing central cholinergic functions, oxidative stress-induced damages, inflammatory responses and neuronal damages in cerebral cortex and hippocampus (Qu et al., 2014). Besides, rutin was capable of modulating neurodegeneration by increasing production of neurotropic factors and attenuating rate of apoptosis (Na et al., 2014). The presence of rutin decreased the acrylamide-induced cytotoxicity, as well as the amount of malondialdehyde, as compared to the control group (Motamedshariaty et al., 2014).

Anti-epileptic functions of hispidulin have been proposed to be one of distinctive characteristics in “Snow lotus”. This chemical was shown to suppress the release of glutamate, activated by a specific K^+^ channel blocker 4-aminopyridine, and which restrained glutamate release from cortical synaptosome *via* presynaptic voltage-dependent Ca^2+^ entry and ERK/synapsin signaling blockage (Lin et al., 2012a). Treatment with hispidulin in cell cultures alleviated the bupivacaine-induced neurotoxicity cell injury *via* enhancing the activations of AMPK and GSK3β levels in mitochondrial membrane (Niu et al., 2014).

### Ischemia/Reperfusion Injury Protection

Acacetin acts as a promising atrium-selective agent in treating atrial fibrillation. In human atrial myocytes, acacetin delayed rectifier K^+^ current and transient outward K^+^ current, as well as prolonging action potential. Besides, acacetin blocked the acetylcholine-activated K^+^ current without affecting other cardiac currents (Li et al., 2008). The water-soluble pro-drug, acacetin phosphate, was reported to protect rats from ischemia/reperfusion injury (**Table 2**). Molecular analysis revealed that acacetin prevented the reduction of anti-oxidative kinases and thioredoxin, and which therefore reduced the release of various inflammatory cytokines. The result of acacetin treatment was to suppress myocyte apoptosis, after induced by ischemia/reperfusion (Liu et al., 2016). In addition, acacetin having a concentration ranging from 0.3 to 3.0 μM was capable of declining cardiomyocyte apoptosis and reactive oxygen species (ROS) production by altering the ratio of Bax/Bcl-2 (Wu et al., 2018). Moreover, acacetin could induce the release of pro-inflammatory cytokines, e.g. TLR-4 and IL-6, in cultured H9C2 cells (Wu et al., 2018).

**Table 2:** Summary of ischemia/reperfusion injury protection of “Snow lotus” and/or its major biochemical.

Models	Parameters measured	Active components	Reference
*In vivo*	Acetylcholine-activated K+ current	Acacetin	Li et al., 2008
*In vivo*	lipid peroxidation activation and infracts size	Rutin	Krishna et al., 2005; Annapurna et al., 2009; Ali et al., 2009
*In vivo*	cGMP, iNOS and 3-NT concentrations	Rutin	Korkmaz & Kolankaya, 2013
*In vitro/ in vivo*	ROS formation and pro-inflammatory cytokine concentration	Acacetin	Wu et al., 2018
*In vitro*	Cytokine levels	Acacetin	Liu et al., 2016

Rutin exhibits similar pharmaceutical functions as that of acacetin (Korkmaz & Kolankaya, 2013; Lv et al., 2018). The pre-treatment of rutin in rats dramatically attenuated cyclic guanosine monophosphate (cGMP) and NO level in serum and inhibited inducible nitric oxide synthase (iNOS) and 3-nitrotyrosine (3-NT) formation in kidney (Korkmaz and Kolankaya, 2013). In addition, the cardio-protective functions of rutin by revealing ischemia–reperfusion-induced myocardial infarction were also reported in rats (Krishna et al., 2005; Ali et al., 2009; Annapurna et al., 2009). The intake of rutin showed the cardio-protection by restraining infract size in normal and diabetic rats: the underling action mechanism was believed to trigger lipid peroxidation in myocardial tissues (Krishna et al., 2005; Ali et al., 2009; Annapurna et al., 2009).

### Adipogenesis Suppression

In high-fat-diet-induced (HFD) obese mice, acacetin reduced body weight and visceral adipose tissue. In differentiated 3T3-L1 cells, applied acacetin increased the level of glycerol in culture medium and significantly inhibited lipid accumulation by Oil Red O staining. In addition, acacetin reduced the transcript and protein expressions of adipogenesis-related transcription factors, including the CCAAT/enhancer-binding protein in cultured adipocytes. In parallel, acacetin treatment increased sirtuin 1 expression and AMPK phosphorylation (Liou et al., 2017). Besides, acacetin was able to suppress the levels of inflammatory mediators, as well as levels of MAPK and NF-κB pathways, in cultured macrophages, as treated with differentiated media deriving from cultured 3T3-L1 adipocytes (Su et al., 2014a; Liou et al., 2017). In insulin-resistant adipocytes, acacetin treatment significantly reduced the levels of chemokines MCP-1 and CCL5 in these cells, as to restore the resistance (Liou et al., 2017).

Administration of rutin in mice could modulate obesity, fatty liver and insulin resistance by increasing energy-consuming gene expressions in brown adipose tissue, including Pgc1α and Dio2, *via* up-regulating mitochondrial size and mitochondrial DNA (mtDNA) content, as well as those mitochondrial biogenesis-related genes, i.e. peroxisome proliferator-activated receptor γ (PARPγ), coactivator-1α (PGC-1α) and nuclear respiratory factor-1 (NRF-1) (Gao et al., 2013; Seo et al., 2015). Furthermore, rutin also restricted the transcriptions of Srebp1c and Cd36, leading to a blockade of developing fatty liver (Gao et al., 2013). After oral administration of rutin in obese mice for 1 week, the rutin-treated group showed over 3-fold longer exhaustive swimming time than the blank control, and which, in parallel, significantly reduced the plasma content of lactic acid (**Table 3**). In rutin-treated mice, the rates of transcription and translation of PGC-1α gene were enhanced in their soleus muscle, and these changes were associated with increased endurance capacity (Su et al., 2014b).

**Table 3:** Summary of adipogenesis suppression effects of “Snow lotus” and/or its major biochemical.

Models	Parameters measured	Active components	Reference
*In vivo/in vitro*	Body and visceral adipose tissue weight; AMPK phosphorylation, MCP-1 and CCL5 contents	Acacetin	Liou et al., 2017
*In vivo/in vitro*	mtDNA content; PARPγ, PGC-1α and NRF-1 transcriptional and translational effects	Rutin	Gao et al., 2013; Seo et al., 2015
*In vivo/in vitro*	Total serum cholesterol, triglyceride and low-density lipoprotein cholesterol and PPARα activations	Hispidulin	Wu and Xu, 2016
*In vivo*	Srebp1c and Cd36 levels	Rutin	Gao et al., 2013
*In vivo*	Exhaustive swimming time	Rutin	Su et al., 2014b
*In vitro*	Inactivation of MAPK and NF-κB pathways	Acacetin	Su et al., 2014a

Hispidulin, another major flavonoid in “Snow lotus”, was shown to possess therapeutic potential in against dyslipidemia. In cultured HepG2 cells, hispidulin acted as an agonist of PPARα and regulated the downstream lipid-metabolizing enzymes, e.g. fatty-acid binding protein 1 and 2, acyl-CoA synthetase 1, carnitine palmitoyltransferase 1α, acetyl-CoA acetyltransferase 1, acyl-coenzyme A dehydrogenase 1 and HMG-CoA synthase 2. In dyslipidemic rat model, the treatment of hispidulin significantly reduced total serum cholesterol, triglyceride and low-density lipoprotein cholesterol, as well as increasing the serum level of high-density lipoprotein cholesterol (Wu and Xu, 2016).

### Anti-Cancer Effect

The anti-cancer properties of “Snow lotus” have been documented. In SK-Hep1 human hepatocellular carcinoma cell line, the cell proliferation was significantly inhibited by the ethanol extract of “Snow lotus” (**Table 4**). This treatment also caused cell cycle arrest at G1-phase, inhibition of DNA synthesis and apoptosis induction *via* caspase 3 and 9 signaling. (Byambaragchaa et al., 2014). In addition, the ethanol extract of “Snow lotus” showed anti-metastatic property by inhibiting invasion and motility of cancer cells *via* activation of an inhibitor for matrix metalloproteinase-2/-9 (MMP-2/-9) (Byambaragchaa et al., 2013). The anti-neoplastic activity of different herbal extracts deriving from “Snow lotus” was shown in PC-3 prostate cancer cells, which included the herbal fractions by using extracting solvents of methanol, ethyl acetate, n-butanol and water. Among these extractives, the ethyl acetate extract showed the most promising effect in inhibiting cancer cell (Yu et al., 2013). More importantly, the ethyl acetate extract of “Snow lotus” markedly reduced the phosphorylation of epidermal growth factor receptor, one of the therapy targets for prostate cancer (Way et al., 2010).

**Table 4:** Summary of anti-cancer functions of “Snow lotus” and/or its major biochemicals.

Models	Parameters measured	Active components	Reference
*In vivo/ in vitro*	Prolong the lifespan of xenograft nude mice	Rutin	Alonso-Castro et al., 2013; Chen et al., 2013
*In vivo*	Capillary-like tube formation and down regulation of VEGF expression	Acacetin	Liu et al., 2011; Bhat et al., 2013
*In vivo*	Suppressing PC-3 cancer and reducing EGFR activation in prostate cancer	“Snow lotus” extract	Way et al., 2010; Yu et al., 2013
*In vitro*	Cell invasion and motility	Rutin Acacetin Hispidulin	Pan et al., 2005; Araújo et al., 2011; Byambaragchaa et al., 2013; Yu et al., 2013; Kim H et al., 2014; Gao et al., 2014; Kim C et al., 2015
*In vitro*	Cell cycle, DNA synthesis	“Snow lotus” extract	Byambaragchaa et al., 2014
*In vivo*	Tumor size and SphK1	Hispidulin and rutin	Lin et al., 2012b; Gao et al., 2017

Acacetin has been shown to induce apoptosis and suppress cell proliferation in gastric carcinoma, oral squamous carcinoma and prostate cancer cells (Pan et al., 2005; Kim H et al., 2014; Kim C et al., 2015). This chemical suppressed the growth of human umbilical vein endothelial cell and the formation of capillary-like tube (Bhat et al., 2013). Acacetin also blocked the phosphorylations of Stat-1 (Tyr701) and Stat-3 (Tyr705), as well as restrained the expression levels of pro-angiogenic factors, e.g. VEGF, eNOS, iNOS, MMP-2 and bFGF, in cancer cells (Bhat et al., 2013). Moreover, over-expression of HIF-1α or AKT inhibited acacetin-restraining VEGF protein level, demonstrating that AKT and HIF-1α could be the downstream key factors in suppressing VEGF expression in ovarian cancer cell (Liu et al., 2011).

Hispidulin is being considered as a potential compound in treating gastric cancer and hepatocellular carcinoma (Yu et al., 2013; Gao et al., 2014). The treatment of hispidulin in AGS human gastric adenocarcinoma cell line could reduce cyclooxygenase-2 (COX-2) expression while keeping a high expression of nonsteroidal-anti-inflammatory-drug-(NSAID-) activated gene-1 (NAG-1). NAG-1 is known to be associated with apoptosis, and the down regulation of this protein expression may promote tumorigenesis (**Table 4**). In NAG-1 constitutive expressed cells, the G1/S phase was arrested, as well as inducing cancer cell apoptosis: this outcome was proposed to be mediated by increased expression of Egr-1 and activated ERK1/2 signaling (Yu et al., 2013). In accordance to anti-cancer notion of hispidulin, Gao and co-workers (Gao et al., 2013) demonstrated that the hispidulin-triggered apoptosis in HepG2 cells could be mediated by the mitochondrial dysfunction, as well as the blockage of P13K/Akt pathway. Subsequently, they showed that hispidulin also prevented proliferation of acute myeloid leukemia cell and triggered cell death *via* an intrinsic mitochondrial pathway by restraining the extracellular matrix metalloproteinase inducer (Gao et al., 2016). Injection of hispidulin in xenograft nude mice in a dose-dependently inhibited tumor size and decreased SphK1 activity, as well as an increase of ceramide accumulation in tumor tissues (Gao et al., 2017).

Excessive anti-cancer functions of rutin have been reported. Rutin dramatically suppressed tumor size justifying anti-leukemic potential in xenograft nude mice (Lin et al., 2012b). Another *in vivo* study has shown that the rutin application reduced detrimental effects and relative organ weight in mice, and more importantly, the increment of mean survival time was observed (Alonso-Castro et al., 2013). *In vitro* study demonstrated the altered Bax/Bcl2 ratio in cultured LAN-5 cell after the treatment of rutin (Chen et al., 2013). Moreover, rutin was known to induce cancer cell apoptosis along with proliferation, angiogenesis and/or metastasis inhibition in colorectal cell lines (Araújo et al., 2011).

### Anti-Inflammation

According to TCM and/or TUM, the extract of “Snow lotus” has been used as a therapeutic agent for inflammation and pain-related disorders. Recent studies have helped to reveal the signaling mechanism contributing in such pharmacological effects. Yi and co-workers had demonstrated that the ethanolic extract of “Snow lotus” possessed anti-inflammatory and anti-nociceptive functions in a mouse model of croton oil-induced ear edema (Yi et al., 2012). In parallel, the oral administration of “Snow lotus” extract was used to treat rats suffering from the collagen II (CII)-induced arthritis (Xu et al., 2016): the infiltration of inflammatory cell, synovial hyperplasia, swelling index and delaying joint destruction were found to be alleviated (**Table 5**). The serum levels of rheumatoid factor, cartilage oligomeric matrix protein (COMP), C-reactive protein and anti-CII IgG antibodies were significantly reduced. Moreover, the therapeutic effects on rheumatoid arthritis by alcohol and water extractives of “Snow lotus” were compared. The effect of ethanolic extract was more potent than that of water extract in treating rheumatoid arthritis in rat model. The ethanolic herbal extract significantly ameliorated rheumatoid arthritis severity, and the over productions of cytokines, e.g. TNF-α, IL-1β, and IL-6, were markedly attenuated in the serum of “Snow lotus” intake rats (Han et al., 2016). Yi and co-workers (2012) presented similar results in *in vitro* analysis of the ethanolic herbal extract: the application of extract in cultures suppressed ROS formation significantly, and the IC50 was 409.6 mg/L. Xiao and co-workers (2011) showed that application of “Snow lotus” extract on cultured macrophage could reduce the levels of PGE2 and NO from 294.9 ng/mL to 238.6 ng/mL and 6.61 ng/mL to 4.52 ng/mL, respectively. Another study illustrated the action of acacetin in mitigating airway hyper-responsiveness in asthmatic mice. The treatment of acacetin reduced the levels of chemokines and Th2-associated cytokines in asthmatic mice, and which effectively suppressed eosinophil infiltration and goblet cell hyperplasia in lung tissue (Huang and Liou, 2012).

**Table 5:** Summary of anti-inflammatory functions of “Snow lotus” and/or its major biochemical.

Models	Parameters measured	Active components	Reference
*In vivo*	Synovial hyperplasia, swelling index, joint destruction and serum cytokine levels	“Snow lotus” and rutin	Chen et al., 2014b; Xu et al., 2016; Han et al., 2016
*In vivo*	Eosinophil infiltration and goblet cell hyperplasia in lung tissue	“Snow lotus” extract	Huang and Liou, 2012
*In vivo*	ROS formation	Rutin	Guardia et al., 2001; Ostrakhovitch and Afanas’ev, 2001
*In vivo*	Skin rush and inflammation Epidermal hyperplasia and protein level	Rutin	Choi et al., 2014; Gęgotek et al., 2017

The pre-treatment of rutin declined the lipopolysaccharide (LPS)-induced arterial blood gas exchange and neutrophils infiltration *in vivo*, and also restrained macrophage inflammatory protein-2 and MMP-9 secretion (Chen et al., 2014b). Significantly decrement in rheumatoid arthritis scores by restraining ROS formation were observed in the rutin-treated rats, and these findings were in line with the rat model of adjuvant arthritis. In addition, rutin inhibited the duplicate inflammatory phases, i.e. acute and chronic stages (Guardia et al., 2001; Ostrakhovitch and Afanas’ev, 2001). Besides, the skin care functions of rutin have been explored to demonstrate the pharmaceutical values of rutin on ultraviolet B (UVB)-induced inflammation *in vivo* (Choi et al., 2014). Pre-treatment and post-treatment of rutin with UVB declined epidermal hyperplasia and protein level in mice (Choi et al., 2014; Gęgotek et al., 2017). Furthermore, rutin suppressed UVB-induced expressions of COX-2 and iNOS through p38 MAP kinase and JNK inhibition.

### Other Pharmaceutical Values

“Snow lotus” is classified as a “kidney-tonifying” in TCM practice, and which is being applied to treat osteoarthritis, articular cartilage injury and osteoporosis with a long history. In a herbal mixture having “Snow lotus” and three other “kidney-tonifying” TCMs, i.e. Astragali Radix, Salviac Miltiorhizae Radix & Rhzizoma and Epimedii Herba, is known to promote the proliferation and osteogenic differentiation of bone marrow-derived mesenchymal stem cells (BMSCs) (Cai et al., 2015). Rutin stimulated proliferation and differentiation of osteosarcoma MG-63 cells by analyzing the increase of cell viability, alkaline phosphatase (ALP), collagen type I and mineralization (Hyun et al., 2014). Similar pharmaceutical functions of rutin have been reported in rat calvarial osteoblast cells (Yang et al., 2006). Osteoclast growth, ROS formation and cytokine secretion were being restricted in the rutin-treated bone cultures (Kyung et al., 2008). Rutin also inhibited the ovariectomy-induced osteopenia in rats by accelerating the formation of osteoblast (Horcajada-Molteni et al., 2000). Hispidulin has estrogenic and anti-osteoporosis functions, and which could act as a promising medicine for osteoporosis modulation. The application of hispidulin in ovariectomy rat model could attenuate bone loss after 2 months of treatment (Zhou et al., 2014). In hispidulin-treated cultures, activated ALP activity was revealed in cultured MC3T3E1 cells, and additionally osteoclastic activity was suppressed in cultured RAW 264.7 cell *via* prohibiting RANKL-induced activation of JNK and p38 (Nepal et al., 2013).

## Conclusion

The pharmaceutical functions of *S. involucrata (*a TUM and/or a TCM herbal material) and its key bioactive chemical ingredients, identified and isolated from this herb, were summarized and reviewed here. These known pharmaceutical properties include immune-modulation, anti-inflammation, adipogenesis inhibition, neuroprotection and ischemia injury protection. Due to its distinct habitat, the resource of “Snow lotus” is rather rare. The plant grows at over 4,000 m altitude in mountainous rocky environment under rather harsh climatic conditions. The plant takes over 8 years to mature before the harvest. Furthermore, over harvesting in China is destroying the growing environment and causing the damage of *S. involucrata* species quality. As a result, these are the major reasons for rarity of “Snow lotus” in the market. The cultivation of “Snow lotus” has been tried by the Xinjiang Technical Institute of Physics & Chemistry in China, the results showed that the harvest period could be shorten from 8 years to 2 years, and the major chemical contents were not decreased. Furthermore, the standard operating procedure of artificial cultivation of “Snow lotus” has been established today, and the cultivation has already extended to 4,000 m altitude. Besides, there are many “Snow lotus” products being available in current herbal market, including cosmeceuticals, food supplements and medications. Although there have been many studies and researches on “Snow lotus”, future research can be carried out in two directions. Firstly, active components, morphological identification, pharmacological effects and metabolisms need to be explored based on the studies of *S. involucrat* and *S. laniceps* (Chen et al., 2014a; Fan et al., 2015; Chen et al., 2016; Yi et al., 2016; Chen et al., 2017). Innovative technology in these areas of research are urgently needed.

## Author Contributions

GG, KC, and KT wrote the main text. JH and TD had great contribution in second time revision, polishing manuscript and helping in revising figures.

## Funding

Supported by Research Fund of Zunyi Medical University for the Doctoral Program (F-937), NNSF of Guangdong (2018A030307074), Science and Technology Planning Project of Guangdong (2014A020221058), China. Shenzhen Science and Technology Innovation Committee (JCYJ20160229205812004; ZDSYS201707281432317; JCYJ20170413173747440; JCYJ20180306174903174). Scientific Research and Innovation of Putian University (2018ZP03, 2018ZP08, 2018ZP07).

## Correction note

A correction has been made to this article. Details can be found at: 10.3389/fphar.2026.1951633.

## Conflict of Interest

The authors declare that the research was conducted in the absence of any commercial or financial relationships that could be construed as a potential conflict of interest.
