# Correction: Saussureae involucratae herba (Snow lotus): review of chemical compositions and pharmacological properties

**DOI:** 10.3389/fphar.2026.1951633

**Published:** 2026-09-11

**Authors:** Guowei Gong, Jing Huang, Yang Yang, Baohui Qi, Guangyi Han, Yuzhong Zheng, Huan He, Kelvin Chan, Karl WK Tsim, Tina TX Dong

**Affiliations:** 1 Department of Biological Engineering, Zunyi Medical University, Zhuhai, China; 2 College of Environmental and Biological Engineering, Putian University, Putian, China; 3 Gansu Institute for Drug Control, Lanzhou, China; 4 Department of Biology, Hanshan Normal University, Chaozhou, China; 5 School of Pharmacy & Biomolecular Sciences, Liverpool John Moores University, Liverpool, United Kingdom; 6 Shenzhen Key Laboratory of Edible and Medicinal Bioresources, HKUST Shenzhen Research Institute, Shenzhen, China; 7 Division of Life Science and Center for Chinese Medicine, The Hong Kong University of Science and Technology, Hong Kong, Hong Kong SAR, China

**Keywords:** saussureae involucratae herba, traditional Chinese medicine, traditional Uyghur medicin, herbal medicine, snow lotus

A correction has been made to the Neuroprotection effect section in page 5.

The original sentence read:

“Acacetin, an o-methylated flavone from “Snow lotus” or other Asteraceae family (Zhao et al., 2016a)…”

The correct citation should read:

“Acacetin, an o-methylated flavone from “Snow lotus” or other Asteraceae family (Zhao et al., 2016b)…”

A correction has been made to the Conclusion section in page 8.

The original sentence read:

“Secondly, in order to extract these active components as much as possible from “Snow lotus”, the potential approaches to pre-treat the extracts could be considered, such as pre-soaking, liquid ammonia pre-treatment and co-digestion (Zhao et al., 2016b; Hassan et al., 2017; Zhao et al., 2017a; Zhao et al., 2017b; Qiao et al., 2018).”

This sentence has been removed in its entirety because the cited references pertain to biomass pretreatment for biofuel production and chemical synthesis, which are not applicable to the extraction of active components from herbal materials.

The corrected paragraph now reads:

“Although there have been many studies and researches on “Snow lotus”, future research can be carried out in two directions. Firstly, active components, morphological identification, pharmacological effects and metabolisms need to be explored based on the studies of S. involucrata and S. laniceps (Chen et al., 2014a; Fan et al., 2015; Chen et al., 2016; Yi et al., 2016; Chen et al., 2017). Innovative technology in these areas of research are urgently needed.”

[Accompanying changes to the Reference List]

To align with the above corrections, the following five references have been removed from the Reference List as they are no longer cited in the article:

Zhao, C., Shao, Q. J., Ma, Z. Q., Li, B., Zhao, X. J. (2016a). Physical and chemical characterizations of corn stalk resulting from hydrogen peroxide presoaking prior to ammonia fiber expansion pretreatment. Ind. Crop Prod. 83, 86–93.

Hassan, M., Umar, M., Ding, W. M., Mehryar, E., Zhao, C. (2017). Methane enhancement through co-digestion of chicken manure and oxidative cleaved wheat straw: Stability performance and kinetic modeling perspectives. Energy. 141, 2314–2320.

Zhao, C., Cao, Y., Ma, Z. Q., Shao, Q. J. (2017a). Optimization of liquid ammonia pretreatment conditions for maximizing sugar release from giant reed (Arundo donax L.). Biomass Bioenerg. 98, 61–69.

Zhao, C., Qiao, X. L., Cao, Y., Shao, Q. J. (2017b). Application of hydrogen peroxide presoaking prior to ammonia fiber expansion pretreatment of energy crops. Fuel. 205, 184–191.

Qiao, X. L., Zhao, C., Shao, Q. J., Hassan, M. (2018). Structural characterization of corn stover lignin after hydrogen peroxide presoaking prior to ammonia fiber expansion pretreatment. Energ Fuel. 32, 6022–6030.

The original version of this article has been updated.

